# Incidence of Neurological Manifestations in Critically Ill COVID-19 Patients: A Low-Resource Setting Experience

**DOI:** 10.7759/cureus.85616

**Published:** 2025-06-09

**Authors:** Sasa Dragic, Andrea Dolencuk, Danica Momcicevic, Biljana Zlojutro, Milka Jandric, Tijana Kovacevic, Vlado Djajic, Pedja Kovacevic, Natasa Milivojevic

**Affiliations:** 1 Medical Intensive Care Unit, University Clinical Centre of the Republic of Srpska and Medical Faculty University of Banja Luka, Banja Luka, BIH; 2 Department of Health, Public Institution, Secondary School Center "Celinac", Celinac, BIH; 3 Department of Neurology, University Clinical Centre of the Republic of Srpska and Medical Faculty University of Banja Luka, Banja Luka, BIH; 4 Neurointensive Care Unit, University Medical Centre Ljubljana, Ljubljana, SVN

**Keywords:** covid-19 pandemic, extrapulmonary manifestations, medical intensive care unit (micu), neurological manifestations in covid-19, sars-cov-2 (severe acute respiratory syndrome coronavirus-2)

## Abstract

Introduction

Severe acute respiratory syndrome coronavirus 2 (SARS-CoV-2) is not primarily a neurotropic virus. However, since angiotensin-converting enzyme 2 (ACE2) receptors are present in the brain, spinal cord, and nerves, the involvement of the nervous system, which could potentially influence the final outcome, cannot be ruled out. This study aimed to assess the incidence and significance of specific neurological manifestations and/or neurological disorders in critically ill COVID-19 patients treated in low-resource settings (LRS), as well as to determine their impact on the clinical outcomes of these patients.

Subjects and methods

The research was designed as a retrospective observational study conducted between October 2020 and February 2021, during the second wave of the COVID-19 pandemic. The incidence of specific neurological manifestations and/or neurological disorders in patients treated at the Medical Intensive Care Unit (MICU) of the University Clinical Centre (UCC) of the Republic of Srpska, Bosnia and Herzegovina, was monitored, and relevant demographic and clinical data were collected. The results were analyzed using methods from descriptive statistics and statistical testing.

Results

Among the 262 patients who met the inclusion criteria, 154 (58.5%) exhibited at least one of the monitored neurological manifestations or neurological disorders. The most frequently observed were impaired consciousness (19.5%), headache (10.2%), myalgia (8%), loss of taste and smell (7.6%), lower back pain (3.4%), and ischemic cerebrovascular stroke (3.4%). All other recorded had an incidence of less than 2%. In terms of clinical outcomes, 128 patients (48.9%) died, 81 (30.9%) were transferred to a step-down unit, and 53 (20.2%) were discharged home. Using Fisher’s exact test, impaired consciousness was found to be significantly associated with a fatal outcome (p = 0.01; OR = 0.33; 95% CI = 0.17-0.64), while acute polyradiculoneuritis was associated with a favorable clinical outcome in the form of discharge home (p = 0.04; OR = 0.96; 95% CI = 0.91-1.01). Headache was more frequently reported in female patients (p = 0.03; OR = 2.51; 95% CI = 1.10-5.80). Impaired consciousness was significantly more frequent in patients aged 51-60 years (p = 0.02; OR = 2.61; 95% CI = 1.12-6.12) and those aged ≥71 years (p = 0.01; OR = 0.36; 95% CI = 0.18-0.73), while epileptic seizures were most commonly observed in the 31-40 age group (p = 0.01; OR = 0.36; 95% CI = 0.18-0.73).

Conclusion

The incidence and significant associations between specific neurological presentations and/or disorders and clinical outcomes, along with observed demographic variations, highlight the importance of comprehensive monitoring of extrapulmonary manifestations (including neurological) in critically ill COVID-19 patients.

## Introduction

The neurological manifestations of COVID-19 in critically ill patients remain under-explored, particularly in low-resource settings (LRS). Although coronaviruses are not primarily neurotropic viruses, angiotensin-converting enzyme 2 (ACE2) receptors, which are crucial for binding the virus to cells and facilitating its internalization, are also present in the brain, spinal cord, and nerves [1]. Severe acute respiratory syndrome coronavirus 2 (SARS-CoV-2) enters cells by its spike protein binding to the ACE2 receptor, followed by proteolytic cleavage of the spike by host proteases - transmembrane protease serine 2 (TMPRSS2) or cathepsin L -which triggers membrane fusion and viral entry. The spike protein is pre-cleaved at the S1/S2 site by furin during virus production, and cleavage at the S2′ site by TMPRSS2 or cathepsin L exposes the fusion peptide necessary for merging viral and cell membranes, allowing viral RNA to enter the host cell [2]. Studies in animal models have demonstrated the passage of the virus through the olfactory epithelium and cribriform plate into the olfactory region of the brain. It is believed that during the viremia phase, the virus can enter nervous tissue due to an impaired blood-brain barrier [3]. In addition to the primary involvement of the nervous system, there is a possibility of immune-mediated damage. Many neurological diseases, such as multiple sclerosis, acute polyradiculoneuritis, chronic inflammatory demyelinating polyneuropathy, and myasthenia gravis, may be aggravated or triggered by the virus, leading to the activation of existing neurological conditions [4].

A wide spectrum of manifestations has been observed in coronavirus infections, with a significant portion involving neurological symptoms and neurological disorders. These can range from mild and reversible to severe cases with serious consequences, significant disability, and even death. Neurological symptoms or disorders may be the primary reason to test a patient for coronavirus infection, and in some cases, they may remain the only signs of the disease (e.g., loss of taste and smell). Furthermore, neurological symptoms or disorders may emerge during the course of treatment, creating uncertainty as to whether they represent complications or additional aspects of the coronavirus infection itself. Several questions emerge, such as whether ischemic stroke is a primary manifestation of the disease or a consequence of heart rhythm disturbances due to extreme dehydration, which may not necessarily be the only cause of arrhythmia. Furthermore, it remains unclear whether venous sinus thrombosis reflects increased blood coagulability caused by SARS-CoV-2 infection or is a result of venous stasis due to prolonged immobility [5].

Currently, the World Bank classifies Bosnia and Herzegovina (including the Republic of Srpska) as an upper-middle-income country (UMIC), but its healthcare system and the issues related to the treatment of critically ill patients are quite similar to those in low- and middle-income countries (LMICs), and accordingly, it can be defined as an LRS [6]. The definition used for LRS in this article refers to healthcare systems in LMICs, as well as UMIC, acknowledging that LRS also exists in high-income countries (HICs) [7].

The main causes of poor outcomes, as well as the lack of prospective studies on critically ill patients in LRS during the COVID-19 pandemic, include limited capacity to manage nonsurgical critically ill patients, a small number of medical intensive care units, an insufficient number of intensive care unit (ICU) beds, a shortage of adequately trained medical personnel, and inadequate academic and research infrastructure [6].

In the Republic of Srpska, one of the two entities in Bosnia and Herzegovina, a significant number of patients were treated at the ICU level 3. This provided valuable experience through the treatment of many critically ill patients, enabling research that monitored the significance of neurological manifestations in this population.

This study aimed to assess the incidence and significance of specific neurological manifestations and/or neurological disorders in critically ill COVID-19 patients treated in LRS. Additionally, the aim of the study was to determine the impact of neurological manifestations and/or neurological disorders on the clinical outcomes of critically ill COVID-19 patients in LRS.

## Materials and methods

Study design

The study was designed as a retrospective observational study conducted from October 2020 to February 2021 (during the second wave of the COVID-19 pandemic) and included critically ill patients of both genders who were treated at the Medical Intensive Care Unit (MICU), University Clinical Centre (UCC) of the Republic of Srpska (ICU level 3). During the specified period, only critically ill COVID-19 patients were treated in this medical (nonsurgical) ICU. This research was approved by the Ethics Committee of the UCC of the Republic of Srpska (decision no. 01-19-178-2/21, dated April 20, 2021).

The MICU serves as a referral center for a region of the Republic of Srpska with approximately 1,000,000 inhabitants and is currently the most advanced multidisciplinary MICU in Bosnia and Herzegovina (and thus in the Republic of Srpska). In this multidisciplinary team, alongside other specialists (pulmonologists, internists, and anesthesiologists) who are also intensivists, a neurologist intensivist was also part of the team, working full-time.

Patient selection

Inclusion criteria were age ≥ 16 years, a positive PCR or rapid antigen test for SARS-CoV-2, and hospital treatment in the MICU (patients with respiratory insufficiency). The exclusion criterion was incomplete medical documentation (missing data on neurological status and clinical outcome and absence of proof of a positive PCR or rapid antigen test for SARS-CoV-2) (n = 23).

Patient intervention

All critically ill patients received standard treatment for COVID-19-associated acute respiratory distress syndrome (ARDS) according to the local hospital protocol, which was derived from guidelines provided by the local Ministry of Health and international recommendations (available evidence-based medicine facts). For certain diagnoses (impairment consciousness, headache, dizziness, loss of taste and smell, epileptic seizure, lower back pain, and muscle pain), anamnestic data or clinical description was sufficient. For the other diagnoses, specific diagnostic criteria were applied. For example, the suspicion of an ischemic cerebrovascular accident or intracerebral hemorrhage was based on neurological examination, while the definitive diagnosis was confirmed by radiological imaging (CT of the brain). The diagnosis of encephalitis and meningitis was clinical in all cases, as we were unable to isolate SARS-CoV-2 from the cerebrospinal fluid despite performing a lumbar puncture, and radiological imaging (CT or MRI of the brain) did not reveal findings suggestive of alternative differential diagnoses. In the case of acute polyradiculoneuritis, a multimodal approach was used by an experienced intensivist neurologist, which included the analysis of anamnestic data, a detailed neurological examination, radiological diagnostics, and cerebrospinal fluid analysis.

Study parameters

During data collection, various medical documents were reviewed, including the initial examination form for COVID-19 patients, medical reports, transfer/discharge letters, diagnostic test results, and patient charts.The following data were recorded: gender, age, clinical outcome (death, discharge to a step-down unit/medical ward, and discharge to home), and neurological manifestations and/or disorders (impairment consciousness, meningitis, encephalitis, ischemic cerebrovascular stroke, intracerebral hemorrhage, subarachnoid hemorrhage, headache, acute polyradiculoneuritis, dizziness, loss of taste and smell, epileptic seizure, lower back pain, and muscle pain).

Statistical analysis

The obtained results were stored in a specially designed MS Excel spreadsheet (Microsoft Corp., Redmond, Washington). Descriptive statistical analysis and hypothesis testing (Fisher's exact test) were performed using IBM SPSS Statistics for Windows, version 26 (IBM Corp., Armonk, NY). Additionally, odds ratios (OR) with 95% confidence intervals (CI) were calculated to measure the strength of associations. A p-value of ≤0.05 was considered statistically significant.

## Results

During the study period, 285 patients were registered, of whom 262 met the inclusion criteria (201 men and 61 women), and 154 (58.5%) of these presented with at least one of the monitored neurological manifestations. In the overall sample, the median age was 61 years, with 79.1% of patients being older than 51 years. The median length of stay in the MICU was eight days, while the median total hospitalization duration was 13 days. Regarding clinical outcomes, 128 patients (48.9%) died, 81 (30.9%) were transferred to the step-down unit, and 53 (20.2%) were discharged home. Table 1 presents data on the incidence and percentage of neurological manifestations and neurological disorders of COVID-19 in the overall sample by gender, including statistical analysis (Fisher's exact test) as well as the calculated OR and corresponding 95% CI.

**Table 1:** Incidence and percentage of neurological manifestations and disorders of COVID-19 by gender with Fisher’s exact test, OR, and 95% CI * p ≤ 0.05. n (%): Total number and percentage; OR: Odds ratio; CI: Confidence intervals.

Parameters	n (%)	Male, n (%)	Female, n (%)	p (OR; 95% CI)
Impaired consciousness	51 (19.5)	38 (14.6)	13 (4.9)	0.73 (1.62; 0.57-2.36)
Meningitis	1 (0.4)	1 (0.4)	0 (0)	1.00 (1.00; 0.98-1.01)
Encephalitis	2 (0.8)	1 (0.4)	1 (0.4)	1.00 (0.99; 0.98-1.00)
Cerebrovascular insult	9 (3.4)	7 (2.6)	2 (0.8)	0.69 (0.40; 0.05-3.28)
Intracerebral hemorrhage	2 (0.8)	1 (0.4)	1 (0.4)	0.41 (3.33; 0.21-54.10)
Subarachnoidal hemorrhage	3 (1.2)	2 (0.8)	1 (0.4)	0.55 (1.66; 0.15-18.61)
Headache	27 (10.3)	16 (6.1)	11 (4.2)	0.03 (2.51; 1.10-5.80)*
Acute polyradiculoneuritis	2 (0.8)	1 (0.4)	1 (0.4)	0.41 (3.33; 0.21-54.10)
Dizziness	4 (1.6)	2 (0.8)	2 (0.8)	0.23 (3.37; 0.46-24.46)
Loss of taste and smell	20 (7.6)	15 (5.7)	5 (1.9)	0.79 (1.11; 0.38-3.18)
Epileptic seizure	3 (1.2)	2 (0.8)	1 (0.4)	0.55 (1.66; 0.19-18.61)
Lower back pain	9 (3.4)	6 (2.2)	3 (1.2)	0.22 (2.75; 0.72-10.58)
Muscle pain	21 (8)	14 (5.3)	7 (2.7)	0.28 (1.73; 0.66-4.51)

Table 2 presents data on the incidence and percentage of neurological manifestations and neurological disorders of COVID-19 in the overall sample by age, including statistical analysis (Fisher's exact test) as well as the calculated OR and corresponding 95% CI.

**Table 2:** Incidence and percentage of neurological manifestations and disorders of COVID-19 by age with Fisher’s exact test, OR, and 95% CI * p ≤ 0.05. n (%): Total number and percentage; OR: Odds ratio; CI: Confidential interval.

Parameters	n (%)	≤18 y, n (%)	p (OR; 95% CI)	18-30 y, n (%)	p (OR; 95% CI)	31-40 y, n (%)	p (OR; 95% CI)	41-50 y, n (%)	p (OR; 95% CI)	51-60 y, n (%)	p (OR; 95% CI)	61-70 y, n (%)	p (OR; 95% CI)	≥71 y, n (%)	p (OR; 95% CI)
Impaired consciousness	51 (19.5)	0 (0)	1.00 (1.24; 1.17-1.32)	1 (0.4)	1.00 (0.97; 0.24-16.12)	3 (1.2)	1.00 (1.14; 0.31-4.11)	2 (0.8)	0.12 (3.29; 0.75-14.38)	7 (2.6)	0.02 (2.61; 1.12-6.12)*	22 (8.4)	0.19 (0.65; 0.25-1.22)	16 (6.1)	0.01 (0.36; 0.18-0.73)*
Meningitis	1 (0.4)	0 (0)	1.00 (1.00; 1.00-1.01)	0 (0)	1.00 (1.00; 0.99-1.01)	0 (0)	1.00 (1.00; 0.99-1.01)	1 (0.4)	1.00 (1.00; 0.99-1.01)	0 (0)	1.00 (1.00; 0.99-1.01)	0 (0)	1.00 (1.00; 0.99-1.02)	0 (0)	0.18 (0.98; 0.94-1.02)
Encephalitis	2 (0.8)	0 (0)	1.00 (1.01; 1.00-1.02)	0 (0)	1.00 (1.00; 0.99-1.02)	0 (0)	1.00 (1.00; 0.99-1.02)	1 (0.4)	1.00 (1.00; 0.99-1.02)	1 (0.4)	1.00 (1.01; 0.99-1.02)	0 (0)	1.00 (0.54; 0.03-8.71)	0 (0)	0.32; 0.21; 0.01-3.41)
Cerebrovascular insult	9 (3.4)	0 (0)	1.00 (1.04; 1.01-1.06)	0 (0)	1.00 (1.04; 1.01-1.06)	0 (0)	1.00 (1.04; 1.01-1.06)	2 (0.8)	1.00 (0.92; 0.11-7.62)	3 (1.2)	0.70 (0.71; 0.17-2.90)	3 (1.2)	0.72 (0.67; 0.17-2.55)	1 (0.4)	1.00 (1.73; 0.21-14.19)
Intracerebral hemorrhage	2 (0.8)	0 (0)	1.00 (1.04; 1.01-1.06)	0 (0)	1.00 (1.01; 0.99-1.02)	1 (0.4)	0.13 (0.07; 0.01-1.09)	0 (0)	1.00 (1.00; 0.99-1.02)	1 (0.4)	1.00 (1.01; 0.99-1.02)	0 (0)	1.00 (0.54; 0.03-8.71)	0 (0)	1.00 (1.00; 0.99-1.02)
Subarachnoidal hemorrhage	3 (1.2)	0 (0)	1.00 (1.01; 1.00-1.03)	0 (0)	1.00 (1.01; 0.99-1.03)	0 (0)	1.00 (1.01; 0.99-1.03)	0 (0)	1.00 (1.01; 0.99-1.03)	1 (0.4)	1.00 (0.71; 0.06-7.99)	1 (0.4)	1.00 (1.08; 0.09-12.11)	1 (0.4)	0.44 (0.42; 0.04-4.74)
Headache	27 (10.3)	1 (0.4)	0.20 (0.11; 0.01-1.84)	1 (0.4)	1.00 (0.92; 0.11-7.76)	2 (0.8)	0.69 (0.86; 0.18-3.96)	2 (0.8)	1.00 (1.49; 0.33-6.69)	8 (3)	0.65 (0.84; 0.35-2.01)	9 (3.4)	1.00 (1.10; 0.47-2.55)	4 (1.6)	1.00 (1.22; 0.40-3.72)
Acute polyradiculoneuritis	2 (0.8)	0 (0)	0.20 (0.11; 0.01-1.84)	0 (0)	1.00 (1.01; 0.99-1.02)	0 (0)	1.00 (1.01; 0.99-1.02)	0 (0)	1.00 (1.00; 0.99-1.02)	2 (0.8)	0.07 (0.97; 0.93-1.01)	0 (0)	0.54 (1.01; 0.99-1.03)	0 (0)	1.00 (1.00; 0.99-1.02)
Dizziness	4 (1.6)	0 (0)	1.00 (1.02; 1.00-1.03)	0 (0)	1.00 (1.02; 1.00-1.03)	0 (0)	1.00 (1.02; 1.00-1.03)	0 (0)	1.00 (1.01; 1.00-1.03)	0 (0)	0.58 (1.02; 1.00-1.04)	4 (1.6)	0.13 (0.18; 0.02-1.71)	0 (0)	0.54 (0.63; 0.06-6.23)
Loss of taste and smell	20 (7.6)	1 (0.4)	0.15 (0.79; 0.01-1.31)	2 (0.8)	0.52 (0.65; 0.08-5.47)	0 (0)	0.38 (1.09; 1.05-1.13)	2 (0.8)	1.00 (1.04; 0.23-4.73)	7 (2.6)	0.43 (0.64; 0.24-1.68)	6 (2.3)	1.00 (1.00; 0.39-2.61)	2 (0.8)	0.54 (2.00; 0.45-8.94)
Epileptic seizure	3 (1.2)	0 (0)	1.00 (1.01; 1.00-1.03)	0 (0)	1.00 (1.01; 0.99-1.03)	1 (0.4)	0.01 (0.03; 0.01-0.36)*	0 (0)	1.00 (1.01; 0.99-1.03)	2 (0.8)	1.00 (0.71; 0.06-7.98)	0 (0)	0.55 (1.02; 0.99-1.04)	0 (0)	1.00 (1.01; 0.99-1.03)
Lower back pain	9 (3.4)	0 (0)	1.00 (1.04; 1.01-1.06)	0 (0)	1.00 (1.04; 1.01-1.06)	2 (0.8)	0.11 (0.22; 0.04-1.15)	0 (0)	0.60 (1.04; 1.01-1.07)	3 (1.2)	1.00 (1.26; 0.26-6.22)	3 (1.2)	0.72 (0.67; 0.17-2.55)	1 (0.4)	1.00 (1.73; 0.21-14.19)
Muscle pain	21 (8)	0 (0)	1.00 (1.09; 1.05-1.13)	0 (0)	1.00 (1.09; 1.05-1.13)	1 (0.4)	1.00 (1.42; 0.18-11.29)	1 (0.4)	0.71 (2.42; 0.31-18.77)	8 (3)	0.45 (0.69; 0.27-1.79)	9 (3.4)	0.24 (0.57; 0.23-1.39)	2 (0.8)	0.55 (2.12; 0.48-9.44

Table 3 presents data on the incidence and percentage of neurological manifestations and neurological disorders of COVID-19 in the overall sample by clinical outcomes, including statistical analysis (Fisher's exact test) as well as the calculated OR and corresponding 95% CI.

**Table 3:** Incidence and percentage of neurological manifestations and disorders of COVID-19 by clinical outcomes with Fisher’s exact test, OR, and 95% CI * p ≤ 0.05. n (%): Total number and percentage; SDU: Step-down unit; OR: Odds ratio; CI: Confidential interval.

Parameters	n (%)	Death, n (%)	p (OR; 95% CI)	Transfer to SDU, n (%)	p (OR; 95% CI)	Discharge home, n (%)	p (OR; 95% CI)
Impaired consciousness	51 (19.5)	36 (13.8)	0.01 (0.33; 0.17-0.64)*	12 (4.6)	0.24 (1.58; 0.78-3.21)	3 (1.2)	0.01 (4.84; 1.44-16.22)*
Meningitis	1 (0.4)	1 (0.4)	1.00 (1.00; 0.99-1.02)	0 (0)	0.31 (0.99; 0.96-1.01)	0 (0)	1.00 (1.00; 0.99-1.01)
Encephalitis	2 (0.8)	2 (0.8)	1.00 (0.97; 0.06-15.67)	0 (0)	1.00 (1.01; 0.99-1.03)	0 (0)	0.36 (0.24; 0.02-3.97)
Cerebrovascular insult	9 (3.4)	6 (2.3)	0.33 (0.47; 0.12-1.93)	3 (1.2)	0.73 (1.59; 0.32-7.82)	0 (0)	0.69 (2.02; 0.25-16.52)
Intracerebral hemorrhage	2 (0.8)	2 (0.8)	0.24 (0.98; 0.96-1.00)	0 (0)	1.00 (1.01; 0.99-1.03)	0 (0)	1.00 (1.01; 0.99-1.02)
Subarachnoidal hemorrhage	3 (1.2)	2 (0.8)	0.62 (0.48; 0.04-5.37)	1 (0.4)	1.00 (0.89; 0.08-10.00)	0 (0)	1.00 (1.01; 0.99-1.03)
Headache	27 (10.3)	9 (3.4)	0.10 (2.07; 0.89-4.79)	10 (3.8)	0.51 (0.74; 0.32-1.70)	8 (3)	0.20 (0.55; 0.23-1.34)
Acute polyradiculoneuritis	2 (0.8)	0 (0)	0.49 (1.01; 0.99-1.04)	0 (0)	1.00 (1.01; 0.99-1.03)	2 (0.8)	0.04 (0.96; 0.91-1.01)*
Dizziness	4 (1.6)	2 (0.8)	0.36 (0.32; 0.03-3.10)	2 (0.8)	1.00 (1.35; 0.14-13.16)	0 (0)	0.59 (1.02; 1.00-1.04)
Loss of taste and smell	20 (7.6)	6 (2.3)	0.10 (2.41; 0.89-6.48)	9 (3.4)	0.21 (0.52; 0.21-1.30)	5 (1.9)	0.56 (0.72; 0.25-2.09)
Epileptic seizure	3 (1.2)	1 (0.4)	1.00 (1.95; 0.17-21.82)	0 (0)	1.00 (0.89; 0.08-10.00)	2 (0.8)	0.49 (0.49; 0.04-5.51)
Lower back pain	9 (3.4)	2 (0.8)	0.17 (3.53; 0.72-17.31)	3 (1.2)	1.00 (0.89; 0.22-3.66)	4 (1.6)	0.08 (0.29; 0.08-1.13)
Muscle pain	21 (8)	10 (3.8)	1.00 (1.07; 0.44-2.62)	3 (1.2)	0.33 (1.99; 0.65-6.13)	8 (3)	0.15 (0.46; 0.17-1.20)

## Discussion

In patients treated at the MICU UCC Republic of Srpska during the second wave of the COVID-19 pandemic, certain neurological manifestations and/or disorders were detected during the initial examination or diagnostic procedures. Some of the neurological manifestations mentioned (such as headache, lower back pain, and muscle pain) can also occur in patients without COVID-19, as part of a general infectious syndrome, elevated body temperature, dehydration, electrolyte imbalance, and so on. Therefore, it is difficult to definitively attribute them as being specific to COVID-19 alone. As COVID-19 is a multisystem disease that affects not only the respiratory system but also frequently involves the cardiovascular, nervous, gastrointestinal, and skin systems, as well as the kidneys, liver, endocrine, and immune systems, it is crucial to understand all of its manifestations, including the neurological ones.

The finding that men are more likely to develop critical illness as part of COVID-19, which requires treatment in ICUs, is also observed in other studies [8-10]. In the study by Jin et al., gender is identified as a risk factor for greater severity and higher mortality in COVID-19 patients, independent of age and overall susceptibility. One possible explanation is that circulating ACE2 levels are higher in men than in women, as well as in patients with diabetes or cardiovascular disease [8]. Additionally, a meta-analysis by Ueyama et al. suggests that male gender may be a predictor of more severe COVID-19 infection [9]. Also, in the study by Grasselli et al., men accounted for 82% of patients [10], which is higher than the proportion in our study, where men represented 76.7% of cases.

When it comes to the age distribution, 79.1% of respondents in our study were older than 61 years. During the study period (the second wave of the COVID-19 pandemic), the vaccine was not yet widely available; therefore, middle-aged and elderly individuals were primarily the patients treated in ICUs for severe forms of pneumonia. The results of other studies show certain variations in terms of age.

For example, in the study by Grasselli et al., the mean age of patients admitted to ICUs was 63 years (the sample included 1,591 patients) [10], whereas in another study involving 136 patients, 61.5% were under 60 years of age [11]. These findings suggest that the capacity and availability of intensive care for all patients (including those who are older and have numerous comorbidities) likely influenced the outcomes.

In the total study sample, 48.9% of patients did not survive; 30.9% of respondents were transferred to another (step-down) ward after their condition improved, and 20.2% of respondents were discharged home. Mortality rates among COVID-19 patients admitted to intensive care units have been reported to range widely, with early studies indicating rates as high as 40%-50%, influenced by factors such as age, comorbidities, and resource availability [10]. According to a study in Italy, which included 1,581 patients who were hospitalized in the intensive care unit, 58% of them were still in the intensive care unit at the time of publication of the results; 16% were discharged from the intensive care unit, and 26% died [12]. The reasons for this heterogeneity in mortality between centers likely stem from differences in the level of development of intensive care medicine in different countries, as well as the availability of resources (space, educated staff, equipment, and medications).

Upon admission to the MICU, initial findings revealed that most patients were adequately conscious, while quantitative impairment of consciousness (somnolence, stupor, and coma) was observed in 19.5% of cases. In our research, it was shown that impaired consciousness at the time of admission to the hospital is associated with higher mortality (p = 0.01; OR = 0.33; 95% CI = 0.17-0.64). As many as 36 out of 51 patients with an initial finding of impaired consciousness ultimately had a fatal outcome. Also, in our study, impaired consciousness was significantly more frequent in patients aged 51-60 years (p = 0.02; OR = 2.61; 95% CI = 1.12-6.12) as well as in those aged ≥71 years (p = 0.01; OR = 0.36; 95% CI = 0.18-0.73).

Chen et al. studied initial symptoms in a cohort of 113 deceased COVID-19 patients and 161 recovered patients, finding that early changes in consciousness occurred significantly more often in those who died (22%) compared to those who recovered (1%) [12]. Mao et al., in a study that included 216 patients, found impaired consciousness in 14.8% of hospitalized patients with a severe form of the disease, compared to 2.4% in those with milder forms of the disease [13].

In our study, only three cases of meningitis and encephalitis were recorded, but without direct isolation of SARS-CoV-2 from the cerebrospinal fluid. Additionally, we did not determine the influence of gender and age on the occurrence of these conditions, nor their impact on clinical outcomes.

Numerous case reports of encephalitis and meningitis have described signs of central nervous system infection with a probable parainfectious etiology (infection-related but not due to direct microbial damage; probably immune-mediated) [14], which was likely the case in our study as well.

Regarding cerebrovascular events, according to the established criteria, we identified ischemic stroke in nine patients (3.4%), subarachnoid hemorrhage in three patients (1.2%), and intracerebral hemorrhage in two patients (0.8%). No significant variations in cerebrovascular events were found in relation to gender, age, or clinical outcome.

In the study by Merkler et al., the incidence of ischemic stroke among critically ill COVID-19 patients with ARDS was reported to be as high as 6%, with COVID-19 recognized as an independent risk factor. The disease induces inflammatory cytokine storms and a hypercoagulable state, both of which increase the risk of thromboembolic complications, particularly in patients with pre-existing vascular risk factors [15].

The data obtained in our study regarding intracerebral hemorrhage showed no correlation with gender, age, or clinical outcome. A possible reason for this is the very low frequency of its occurrence in the overall sample. The study by Kanani et al. based on autopsy findings showed that nontraumatic brain hemorrhage (particularly intracerebral hemorrhage and subarachnoid hemorrhage) plays a critical role in sudden and unexpected deaths, especially in overweight or obese individuals, highlighting the need for vigilance toward fatal cerebrovascular events such as potential post-COVID complications in the current post-pandemic era [16].

In our study, headache - one of the better-known neurological symptoms of COVID-19 - proved to be very prevalent in the examined sample, occurring in 10.3% of subjects. It was statistically significantly more frequent in women (p = 0.03; OR = 2.51; 95% CI = 1.10-5.80), although it did not have a statistically significant influence on the patient's clinical outcome.

In the study by Uygun et al., the prevalence of headache in COVID-19 patients was 10.9%, which is similar to our findings [17]. Bolay et al. stated that COVID-19 patients reported headache as an initial symptom in approximately 6%-10% of cases and that the possible pathophysiological mechanisms of such headache include activation of peripheral trigeminal nerve endings either directly by the SARS-CoV-2 virus or indirectly through vasculopathy and/or increased levels of circulating pro-inflammatory cytokines and hypoxia [18]. In the case series by Toptan et al., headache associated with COVID-19 is described as typically acute in onset and usually differing in character from previous headaches [19]. All of these are valuable findings that highlight headache as a symptom that should not be overlooked in COVID-19 patients.

When it comes to acute polyradiculoneuritis, two cases were identified in this study - one male and one female. In both cases, the patients were discharged home in a recovered state. Using Fisher's exact test, we demonstrated that patients with acute polyradiculoneuritis had a statistically significantly higher probability of a positive treatment outcome (discharge home) (p = 0.04; OR = 0.96; 95% CI = 0.91-1.01).

In the study by Toscano et al., five cases of acute polyradiculoneuropathy were reported across three hospitals in northern Italy during the COVID-19 outbreak. Three of the patients required mechanical ventilation, and two of them achieved complete recovery following treatment with immunoglobulins [20]. The case presentation by Sedaghat and Karimi shows that acute polyradiculoneuritis should be considered a neurological complication of COVID-19, as respiratory involvement is common in COVID-19, and SARS-CoV-2 may be a risk factor for the development of acute polyradiculoneuritis [21].

Dizziness, as a neurological symptom, was found in 1.5% of the respondents in this study, with no significant difference in incidence between sexes or age groups and no impact on the clinical outcome. The report by Saniasiaya et al. provides data on the frequency of vertigo, which is quite heterogeneous, and states that vertigo is rarely treated as a separate symptom [22]. Another fairly common symptom involving the central nervous system (CNS) is a disorder of the sense of taste and smell, which appeared in 7.6% of respondents in this study, with no significant influence of gender or age on the occurrence of these symptoms, nor on the clinical outcome.

In the study by Lee et al., where 3,191 patients were surveyed by telephone in Korea, acute loss of smell and taste was reported in 15.3% of patients during the early phase of COVID-19 and in 15.7% of those with asymptomatic to mild disease. In that study, most patients with loss of taste and smell recovered within three weeks, with a median recovery time of seven days for both symptoms [23]. These findings suggest that SARS-CoV-2 affects the olfactory region, a phenomenon also demonstrated in animal models by Jackson et al. [2].

In our study, epileptic seizures were recorded in three patients as the first sign of the disease, with a statistically higher frequency of seizures in the age group 31-40 years (p = 0.01; OR = 0.36; 95% CI = 0.18-0.73), while no association was found between epileptic seizures and gender or clinical outcomes.

In the retrospective study by Lu et al. conducted on 304 hospitalized COVID-19 patients, no cases of new-onset acute symptomatic seizures or status epilepticus were observed, suggesting that seizures are a rare neurological complication of COVID-19 [24]. A retrospective case series by Anand et al. involving 1,043 subjects, conducted in 2020, revealed an incidence of epileptic seizures of 0.7% among COVID-19 patients [25]. In the study by Cho and Kim, among 1,487 hospitalized patients with COVID-19, six patients (0.4%) developed new-onset epileptic seizures during hospitalization. All six patients were admitted to the intensive care unit, accounting for approximately 3.6% of all ICU admissions during the study period [26].

Lower back pain and muscle pain were among the most frequent neurological manifestations recorded in this study. In this study, 3.5% of patients had lower back pain, and 8% had muscle pain. However, no significant difference between genders or age groups was found for either manifestation nor was there a significant influence of these manifestations on the clinical outcome.

In the article by Widyadharma et al., muscle pain is highlighted as one of the most frequent symptoms associated with COVID-19, with reported prevalence in studies ranging from 11% to 50%. The symptom tends to be more common in patients with severe clinical presentations. Potential underlying mechanisms include the body’s systemic inflammatory response to infection, cytokine storm, and direct muscle injury mediated by ACE-2 receptors, which are expressed in muscle tissue [27]. Muscle pain is not limited to patients with COVID-19 but is also a common manifestation in patients hospitalized in intensive care units for other reasons. In the scoping review by Gustafson et al., it is noted that patients who spend prolonged periods in the ICU, including those without COVID-19, frequently experience muscle pain due to extended immobilization, sedative use, and the nonspecific effects of intensive treatment. Muscle pain and weakness are common in the post-ICU population, with studies suggesting that up to 60% of patients may suffer from symptoms such as muscle pain and reduced muscle function several months after ICU discharge. These symptoms can significantly affect patients' quality of life and recovery [28].

The primary limitations of this study are inherent to its retrospective design, which, despite a relatively large sample size, limited the detection of less frequent neurological manifestations and diagnoses. Moreover, despite considerable effort, distinguishing discrete neurological symptoms from those resulting from pre-existing neurological conditions or the systemic effects of a generalized infectious syndrome remained a methodological challenge.

## Conclusions

This study demonstrated that the most common neurological manifestations in critically ill COVID-19 patients during the second wave of the pandemic were impaired consciousness (19.5%), headache (10.3%), muscle pain (8%), loss of taste and smell (7.6%), lower back pain (3.4%), and ischemic cerebrovascular insult (3.4%). Other observed neurological symptoms, such as meningitis, encephalitis, intracerebral and subarachnoid hemorrhage, acute polyradiculoneuritis, dizziness, and epileptic seizures, were present in less than 2% of cases.

A significant association was observed between impaired consciousness and fatal outcomes, while acute polyradiculoneuritis was statistically more likely in patients with a good outcome who were discharged home. Headache was more frequently reported among female patients, impaired consciousness was more common in individuals aged 51-60 and ≥71 years, and epileptic seizures were predominantly observed in the 31-40 age group. Finally, many unknowns remain, particularly regarding the spectrum of non-pulmonary manifestations of COVID-19, and further research is essential to uncover all aspects of this disease, with this study highlighting the need for heightened neurological surveillance in COVID-19 patients, especially in LRS.

## References

[1] Maury A, Lyoubi A, Peiffer-Smadja N, de Broucker T, Meppiel E (2021). Neurological manifestations associated with SARS-CoV-2 and other coronaviruses: a narrative review for clinicians. Rev Neurol (Paris).

[2] Jackson CB, Farzan M, Chen B, Choe H (2022). Mechanisms of SARS-CoV-2 entry into cells. Nat Rev Mol Cell Biol.

[3] Butowt R, von Bartheld CS (2021). Anosmia in COVID-19: underlying mechanisms and assessment of an olfactory route to brain infection. Neuroscientist.

[4] Collantes ME, Espiritu AI, Sy MC, Anlacan VM, Jamora RD (2021). Neurological manifestations in COVID-19 infection: a systematic review and meta-analysis. Can J Neurol Sci.

[5] Baig AM, Sanders EC (2020). Potential neuroinvasive pathways of SARS-CoV-2: Deciphering the spectrum of neurological deficit seen in coronavirus disease-2019 (COVID-19). J Med Virol.

[6] Kovacevic P, Djajic V, Momcicevic D (2023). Boosting ICU capacity during the COVID-19 pandemic in the Western Balkan region, the Republic of Srpska experience. J Public Health Res.

[7] Losonczy LI, Papali A, Kivlehan S (2021). White paper on early critical care services in low resource settings. Ann Glob Health.

[8] Jin JM, Bai P, He W (2020). Gender differences in patients with COVID-19: focus on severity and mortality. Front Public Health.

[9] Ueyama H, Kuno T, Takagi H (2020). Gender difference is associated with severity of coronavirus disease 2019 infection: an insight from a meta-analysis. Crit Care Explor.

[10] Grasselli G, Zangrillo A, Zanella A (2020). Baseline characteristics and outcomes of 1591 patients infected with SARS-CoV-2 admitted to ICUs of the Lombardy Region, Italy. JAMA.

[11] Liu Y, Mao B, Liang S (2020). Association between age and clinical characteristics and outcomes of COVID-19. Eur Respir J.

[12] Chen T, Wu D, Chen H (2020). Clinical characteristics of 113 deceased patients with coronavirus disease 2019: retrospective study. BMJ.

[13] Mao L, Jin H, Wang M (2020). Neurologic manifestations of hospitalized patients with coronavirus disease 2019 in Wuhan, China. JAMA Neurol.

[14] Ahmad I, Rathore FA (2020). Neurological manifestations and complications of COVID-19: a literature review. J Clin Neurosci.

[15] Merkler AE, Parikh NS, Mir S (2020). Risk of ischemic stroke in patients with coronavirus disease 2019 (COVID-19) vs patients with influenza. JAMA Neurol.

[16] Kanani J, Sheikh MI (2025). Exploring nontraumatic brain hemorrhage in sudden and unexpected deaths: a novel autopsy-based investigation. Asian J Neurosurg.

[17] Uygun Ö, Ertaş M, Ekizoğlu E (2020). Headache characteristics in COVID-19 pandemic-a survey study. J Headache Pain.

[18] Bolay H, Gül A, Baykan B (2020). COVID-19 is a real headache!. Headache.

[19] Toptan T, Aktan Ç, Başarı A, Bolay H (2020). Case series of headache characteristics in COVID-19: headache can be an isolated symptom. Headache.

[20] Toscano G, Palmerini F, Ravaglia S (2020). Guillain-Barré syndrome associated with SARS-CoV-2. N Engl J Med.

[21] Sedaghat Z, Karimi N (2020). Guillain Barre syndrome associated with COVID-19 infection: a case report. J Clin Neurosci.

[22] Saniasiaya J, Kulasegarah J (2021). Dizziness and COVID-19. Ear Nose Throat J.

[23] Lee Y, Min P, Lee S, Kim SW (2020). Prevalence and duration of acute loss of smell or taste in COVID-19 patients. J Korean Med Sci.

[24] Lu L, Xiong W, Liu D (2020). New onset acute symptomatic seizure and risk factors in coronavirus disease 2019: a retrospective multicenter study. Epilepsia.

[25] Anand P, Al-Faraj A, Sader E (2020). Seizure as the presenting symptom of COVID-19: a retrospective case series. Epilepsy Behav.

[26] Cho YJ, Kim HK (2022). New-onset seizures in patients with COVID-19: a case series from a single public hospital in Korea. J Korean Med Sci.

[27] Widyadharma IP, Sari NN, Pradnyaswari KE (2020). Pain as clinical manifestations of COVID-19 infection and its management in the pandemic era: a literature review. Egypt J Neurol Psychiatr Neurosurg.

[28] Gustafson OD, Williams MA, McKechnie S, Dawes H, Rowland MJ (2021). Musculoskeletal complications following critical illness: a scoping review. J Crit Care.

